# Memantine in the management of a clinically challenging case of bipolar disorder

**DOI:** 10.4103/0019-5545.49455

**Published:** 2009

**Authors:** Vivek Agarwal, Adarsh Tripathi

**Affiliations:** Department of Psychiatry, CSM Medical University UP (Formerly K. G. Medical University), Lucknow - 226 003, India

**Keywords:** Bipolar disorder, memantine, treatment resistant, treatment intolerance

## Abstract

Use of memantine in bipolar disorders is not been studied except one case report. We report a case in which use of memantine lead to better medication tolerance and improvement in symptoms in bipolar disorder.

## INTRODUCTION

Memantine is a well known cognitive enhancer in dementia. However, its role in the treatment of bipolar disorder has not been studied except one case report.[1] We present a case of bipolar disorder that showed treatment resistance as well as intolerance to psychotropic medications. He showed idiopathic bilateral frontotemporal atrophy on CT scan head and his symptoms improved after augmentation with memantine.

## CASE REPORT

A 42 year old male was hospitalized in March 2007 with symptoms of increased talkativeness, religiosity, motor activity and irritability, big talks and decreased sleep for 7 days. The young mania rating scale (YMRS)[2] sore was 41. He was diagnosed as bipolar affective disorder current episode mania (ICD-10, WHO, 1992). He had history of multiple episodes of mania and depression in the past 27 years and was hospitalized multiple times. In the past episode he improved and was maintained on sodium valproate 1000 mg/day, lithium Carbonate 900 mg/day and chlorpromazine 400 mg/day for 2 years. However, in past 6 months he was taking medications irregularly.

The patient was started on the sodium valproate1000 mg/day, olanzapine 20 mg/day and lorazepam 6 mg/day, which were increased up to 1500mg/d, 30 mg/d and 6 mg/day respectively after 1 week as there was no improvement. However, within 2 days he became delirious. All the medications were stopped and general care was provided. His general condition improved in 5 days and manic symptoms including severe agitation and aggression returned. He was given Quetiapine (600mg/day) and oxcarbamazepine (400 mg/day) for 2 weeks but his symptoms worsened. Therefore he was given 1000 mg/day sodium valproate along with Lithium (900 mg/day) and chlorpromazine (800 mg/day). On this he again became delirious in 7 days. A specific intolerance to benzodiazepine was also noted.

The patient's hematological, serum electrolyte, liver and kidney function test and thyroid hormone levels were normal. However, CT scan of brain showed bilateral frontotemporal atrophy. In neurologist's opinion the atrophy was not clinically significant. The reason or the duration of atrophy could not be found.

Now he was started on sodium valproate 1000mg/day and clozapine 25 mg/day which were gradually increased up to 1500mg/day, 300 mg/day respectively. After this treatment he showed marked improvement in 4 weeks (YMRS score 10). However, his symptoms again started increasing despite good compliance. On further increasing the valproate to 2000 mg/day he became disoriented next day, so it was decreased to 1500 mg/day. On increasing clozapine to 350 mg/day he became very drowsy, so it was decreased to 300mg/day. Then 15 mg/day aripiprazole was added but he did not show any improvement in 2 weeks. In view of the CT scan findings memantine was added 10 mg/day and aripiprazole was stopped. After 2 weeks it was possible to increase clozapine to 350 mg/day. Complete remission of symptoms occurred in next 4 weeks. He was also assessed for cognitive functions but no significant decline was observed. He remained in regular follow-up and is well for last one year.

## DISCUSSION

In our patient, use of memantine lead to significant increase in tolerance to medication and it was possible to give multiple medications in adequate doses. Memantine also helped in the mood stabilization as the symptoms worsened even on clozapine treatment. In a case report, memantine was found useful in the treatment of bipolar disorder.[1] Although, the frontotemporal atrophy was clinically not significant but in our opinion this may be a possible reason for intolerance as well as resistance to medication in our patient. Few previous studies have reported deep subcortical white matter lesions to be associated with poor outcome in bipolar disorder[3] while our patient had cortical lesion.

Further studies should be done to look for the reasons of treatment intolerance or resistance as well as the efficacy of memantine augmentation in treatment resistant bipolar disorder.
